# A multi-method approach to modeling COVID-19 disease dynamics in the United States

**DOI:** 10.1038/s41598-021-92000-w

**Published:** 2021-06-14

**Authors:** Amir Mokhtari, Cameron Mineo, Jeffrey Kriseman, Pedro Kremer, Lauren Neal, John Larson

**Affiliations:** Booz Allen Hamilton, 4747 Bethesda Ave., Bethesda, MD 20814 USA

**Keywords:** Viral infection, Computational models

## Abstract

In this paper, we proposed a multi-method modeling approach to community-level spreading of COVID-19 disease. Our methodology was composed of interconnected age-stratified system dynamics models in an agent-based modeling framework that allowed for a granular examination of the scale and severity of disease spread, including metrics such as infection cases, deaths, hospitalizations, and ICU usage. Model parameters were calibrated using an optimization technique with an objective function to minimize error associated with the cumulative cases of COVID-19 during a training period between March 15 and October 31, 2020. We outlined several case studies to demonstrate the model’s state- and local-level projection capabilities. We further demonstrated how model outcomes could be used to evaluate perceived levels of COVID-19 risk across different localities using a multi-criteria decision analysis framework. The model’s two, three, and four week out-of-sample projection errors varied on a state-by-state basis, and generally increased as the out-of-sample projection period was extended. Additionally, the prediction error in the state-level projections was generally due to an underestimation of cases and an overestimation of deaths. The proposed modeling approach can be used as a virtual laboratory to investigate a wide range of what-if scenarios and easily adapted to future high-consequence public health threats.

## Introduction

During the current COVID-19 pandemic, global efforts have taken place to contain the spread of the virus and develop effective non-therapeutic (e.g., social distancing, partial and full lockdowns) and therapeutic treatments (e.g., vaccination). As the COVID-19 pandemic has spread across the globe since early 2020, researchers have identified gaps in data and our understanding of ways in which the disease spreads within and between communities including its potential impacts on general and at-risk populations^1,2^.

Computational modeling has been long employed to further increase our understanding of complex infectious diseases as well as their development, spread dynamics, and potential treatments^3^. Using computational modeling, we have been able to identify common patterns in infectious diseases allowing us to leverage lessons learned through investigating past widespread disease events to predict who may get infected, where vaccination efforts should be prioritized, and how to limit the spread of infectious diseases in future events^4–7^.

Two methods, System Dynamics (SD) and Agent-Based Modeling (ABM), have been frequently used in recent years to investigate the complex nature of infectious diseases and their potential containment strategies. SD has a long history of being applied to the study of infectious disease epidemiology. This method operates at a high level of abstraction by compartmentalizing the population into different disease stages such as Susceptible (S), Infected (I), and Recovered (R), among others while assuming population homogeneity within each compartment^8,9^. Previous studies have identified limitations of SD in modeling infectious diseases such as inability to model multi-strain infections, deterministic nature, inability to model time-varying infectivity, and assumptions regarding population homogeneity, among others^10^. With the boom in computer processing capability in the twenty-first century, ABM has been recently used in modeling infectious disease dynamics^11,12^. ABM uses a bottom-up approach, where a complex dynamic system is described as interacting objects with their own behaviors such that systemic behavior can potentially emerge as a summary of the individual actions of agents^13,14^. ABM for infectious diseases focuses on incorporating individual information such as personal interactions, movements, and health information in an attempt to provide a more granular profile of disease spread as compared to the homogenous population of SD models. However, ABM is not without its limitations: (1) model parameters (e.g., reproduction number for infectious diseases) are often difficult to quantify; (2) model validation can be difficult to assess, particularly when modeling unobserved associations^15^; (3) ABM can become exceedingly computationally intensive when applied to large populations^16^; and (4) lack of individualized data may result in increased model assumptions and uncertainty^17^.

In this paper, we propose a multi-method, also known as hybrid, modeling approach to community-level infectious disease spread. The idea of multi-method modeling is to integrate different methods of computational modeling to overcome the limitations of individual methods and get the most from each one^18–20^. Our Multi-Method Community Disease Risk Model, hereafter referred to as M^2^-CDRM, combines the advantages of SD and ABM, allowing the simulation of spatially explicit scenarios representing future states of disease transmission within different communities and testing risk management policies across a wide range of scenarios using what-if analysis. The model integrates multiple layers of data including population demographics, observed cases of illness and death, and hospital demands at the local county-level within different states to make location-specific predictions about COVID-19 illness and death. M^2^-CDRM can be used as a virtual laboratory to: (1) identify “hot spots” of potential areas (e.g., counties) with highest levels of infected individuals within the United States that can potentially act as infection hubs during the ongoing pandemic; (2) examine population-specific characteristics (e.g., gender, age) that can result in disproportionate distribution of mortality and morbidity in cases across the United States; (3) prioritize counties based on their perceived disease risks considering multiple decision criteria; and (4) evaluate the effectiveness of candidate mitigation options (e.g., social distancing, wide-spread testing) aimed at reducing the likelihood of disease transmission within different communities. This paper outlines a case study of our proposed approach focused on modeling COVID-19 at a community level in the United States. Additionally, we introduced an example of how this model could be potentially used in conjunction with a Multi-Criteria Decision Analysis (MCDA) framework to assess and prioritize different communities in terms of their perceived risk of COVID-19.

## Methods

### Model overview

**Figure 1:**
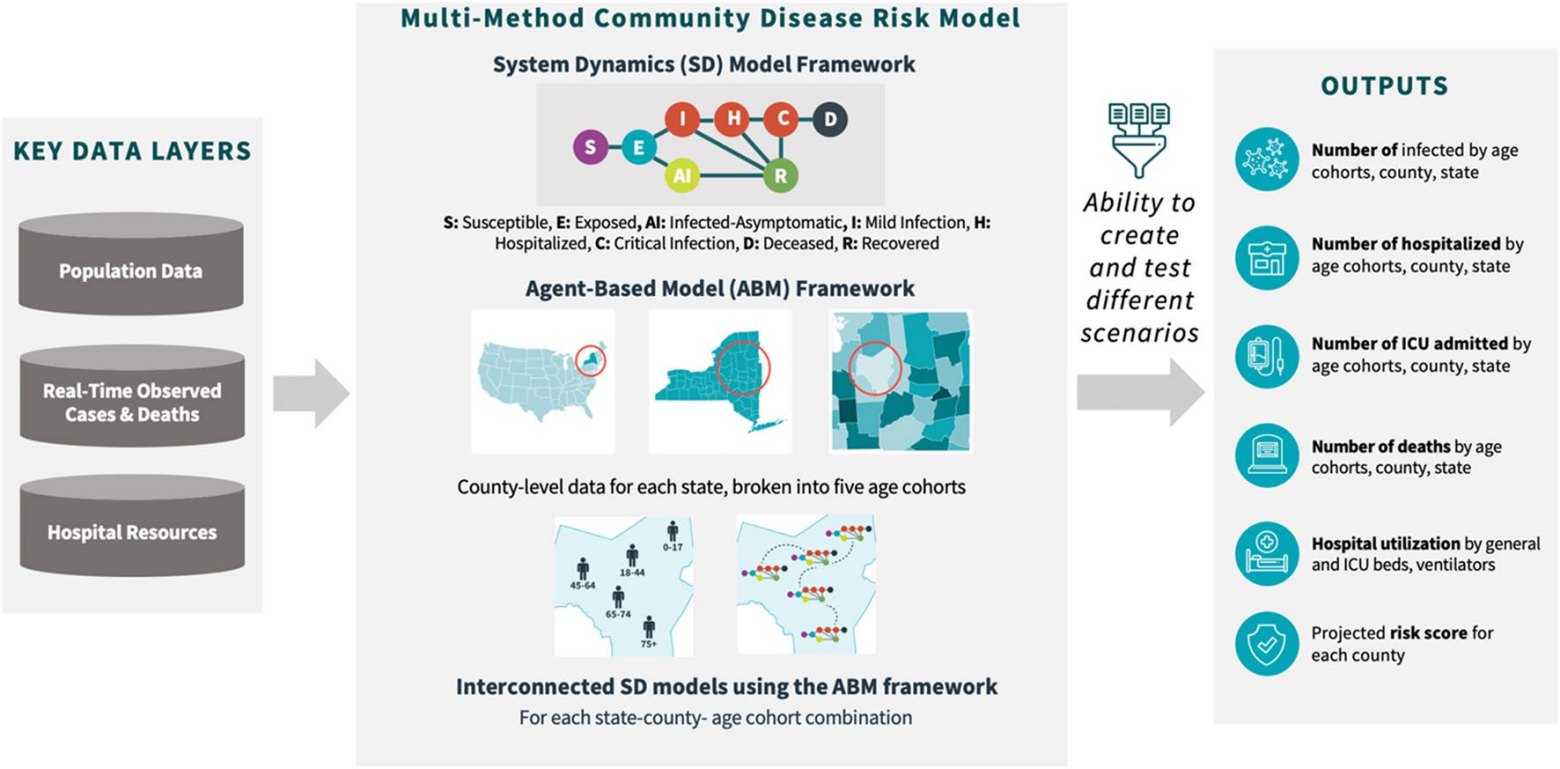
Overview of the multi-method community disease risk model (M^2^-CDRM) including key data layers, modeling framework, and model outputs.

We developed M^2^-CDRM as a highly customizable, evidence-based, and data-driven model by integrating an SD modeling approach within an ABM framework to study the COVID-19 transmission on multiple levels of aggregation in the United States (Fig. 1). The model is implemented in AnyLogic (Professional Edition, Version: 8.5.2, Link: https://www.anylogic.com), a modeling framework that integrates support for SD, ABM, and other dynamic computational methods. M^2^-CDRM included all 50 states as well as their individual counties with a simulation period between March 15 and December 31st, 2020.

### Disease transmission models

With COVID-19, different subpopulations have been shown to be more or less susceptible, more or less likely to be infectious, and more or less likely to recover from the disease^21–23^. Therefore, treating the entire population with the same static assumptions about these rates can cause decision makers to miss key aspects of the disease’s likely trajectory. M^2^-CDRM addresses this limitation by including five separate SD models to simulate COVID-19 disease dynamics in distinct age cohorts within each individual county: 0–17, 18–44, 45–64, 65–74, and 75 + years of age. While these cohorts were initially selected to stratify the population based on their ages, the model design is flexible and can accommodate any age stratification. Each SD model was defined using eight compartments, including Susceptible (S), Exposed (E), Asymptomatic Infection (AI), Mild Infection (MI), Severe Infection (SI), Critical Infection (CI), Recovered (R), and Death (D). In each model, severe infection and critical infection represented general admission to the hospital as well as ICU admission, respectively. During the early stage of the pandemic, the confirmed COVID-19 case counts in the U.S. did not capture the total burden of the pandemic. This was primarily because testing was restricted to individuals with moderate to severe symptoms due to limited test availability^24^. Therefore, in order to correct for biased testing and imperfect diagnostic accuracy and provide a more realistic assessment of COVID-19 burden, we further adjusted simulated infection cases (I) by an under-reporting factor.

**Figure. 2:**
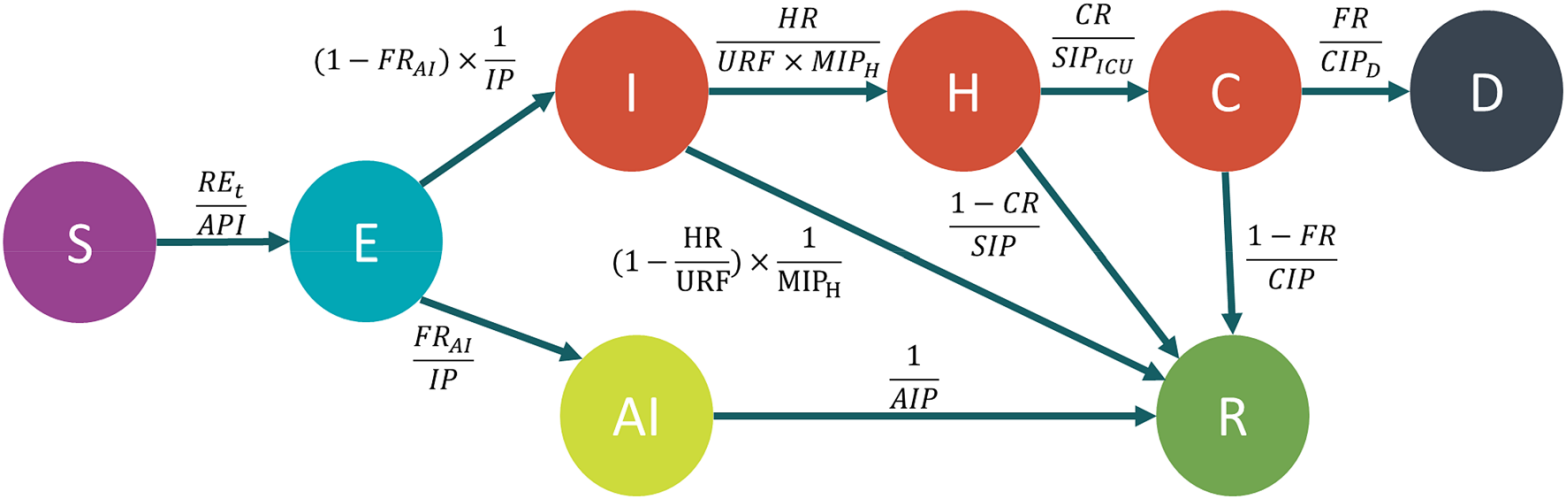
Disease transmission model including Susceptible (S), Exposed (E), Infected (I), Asymptomatic Infection (AI), Hospitalization (H), Critical Infection (C), Recovery (R), and Death (D) stages.

For each state, the spread of COVID-19 in county *j* and for age cohort *i* was modeled based on the following set of differential equations (Fig. 2):

1 $$\frac{{dS_{i,j} }}{dt} = - \frac{{S_{i,j} \times \mathop \sum \nolimits_{i = 1}^{5} (I_{i,j} + AI_{i,j} )}}{{S_{j} \left( 0 \right) - D_{j} }} \times \frac{{RE_{t,j} }}{{API_{i} }}$$

2 $$\frac{{dE_{i,j} }}{dt} = \frac{{S_{i,j} \times \mathop \sum \nolimits_{i = 1}^{5} (I_{i,j} + AI_{i,j} )}}{{S_{j} \left( 0 \right) - D_{j} }} \times \frac{{RE_{t,j} }}{{API_{i} }} - \frac{{E_{i,j} }}{IP}$$

3 $$\frac{{dI_{i,j} }}{dt} = \left( {1 - FR_{AI} } \right) \times \frac{{E_{i,j} }}{IP} - \frac{{I_{i,j} }}{{MIP_{H} }} \times \frac{{HR_{i} }}{URF} - \frac{{I_{i,j} }}{MIP} \times \left( {1 - \frac{{HR_{i} }}{URF}} \right)$$

4 $$\frac{{dAI_{i,j} }}{dt} = FR_{AI} \times \frac{{E_{i,j} }}{IP} - \frac{{AI_{i,j} }}{AIP}$$

5 $$\frac{{dH_{i,j} }}{dt} = \frac{{I_{i,j} }}{{MIP_{H} }} \times \frac{{HR_{i} }}{URF} - \frac{{H_{i,j} }}{{SIP_{ICU} }} \times CR_{i} - \frac{{H_{i,j} }}{SIP} \times (1 - CR_{i} )$$

6 $$\frac{{dC_{i,j} }}{dt} = \frac{{H_{i,j} }}{{SIP_{ICU} }} \times CR_{i} - \frac{{C_{i,j} }}{{CIP_{D} }} \times FR_{i} - \frac{{C_{i,j} }}{CIP} \times \left( {1 - FR_{i} } \right)$$

7 $$\frac{{dD_{i,j} }}{dt} = \frac{{C_{i,j} }}{{CIP_{D} }} \times FR_{i}$$

8 $$\frac{{dR_{i,j} }}{dt} = \frac{{I_{i,j} }}{MIP} \times \left( {1 - \frac{{HR_{i} }}{URF}} \right) + \frac{{AI_{i,j} }}{AIP} + \frac{{H_{i,j} }}{SIP} \times \left( {1 - CR_{i} } \right) + \frac{{C_{i,j} }}{CIP} \times \left( {1 - FR_{i} } \right)$$

9 $$API_{i} = \frac{1}{{\frac{1}{MIP} \times \left( {1 - \frac{{HR_{i} }}{URF}} \right) + \frac{1}{{MIP_{H} }} \times \left( {\frac{{HR_{i} }}{URF}} \right)}} \times \left( {1 - FR_{AI} } \right) + AIP \times FR_{AI}$$

where *S*_*i,j*_ represents susceptible population in age cohort *i* (*i* = 1,…,5) in county *j*, *S*_*j*_*(0)* represents initial susceptible population in county *j* across all age cohorts, *E*_*i,j*_ represents exposed population in age cohort *i* in county *j*, *I*_*i,j*_ represents symptomatic infectious population in age cohort *i* in county, *AI*_*i,j*_ represents asymptomatic infectious population in age cohort *i* in county *j, H*_*i,j*_ represents hospitalized population (severe infection) in age cohort *i* in county *j*, *C*_*i,j*_ represents critically infected population (ICU admission) in age cohort *i* in county *j*, *R*_*i,j*_ represents recovered (non-infectious) population in age cohort *i* in county *j, D*_*i,j*_ represents deceased population in age cohort *i* in county *j, IP* represents incubation period (days), *FR*_*AI*_ represents fraction of asymptomatic population, *MIP*_*H*_ represents duration of mild infection prior to hospitalization (days), *MIP* represents duration of mild infection prior to recovery (days), *AIP* represents duration of asymptomatic infection (days), *HR*_*i*_ represents hospitalization rate for age cohort *i* (*i* = 1,…,5), *URF* represents under-reporting factor of symptomatic infections, *SIP*_*ICU*_ represents severe infection period prior to transfer to ICU (days), *SIP* represents severe infection period prior to recovery (days), *CR*_*i*_ represents critical infection rate in age cohort *i* (*i* = 1,…,5), *CIP*_*D*_ represents critical infection period prior to death (days), *CIP* represents critical infection period prior to recovery (days), FR_i_ represents fatality rate in age cohort *i* (*i* = 1,…,5), *API*_*i*_ represents average period of infectiousness in age cohort *i* (*i* = 1,…,5), and *RE*_*t,j*_ represents effective reproduction number at time *t* in county *j*.

### ABM framework to connect SD models

Within each county, we defined population age cohorts (0–17, 18–44, 45–64, 65–74, 75 +) as individual agents. Each of these individual agents was then coupled with all other agents within the same county with explicit interactivity patterns. By focusing on micro-level interactions, this framework was able to explain emergent patterns such as transient dynamics on a system level and identify important mechanisms, taking into account heterogeneity of entities (e.g., individual age cohorts as agents) and spatial and temporal heterogeneity of processes (e.g., variability in disease dynamics across different counties). Additionally, the ABM structure allowed for the possibility of advanced data inputs such as age-specific reproduction numbers, interaction, and mobility patterns across age cohorts and counties, county- and age-specific adherence to social distancing policies, and what-if analysis such as customizable vaccine distribution networks. Outputs from our framework were timeseries of system-level variables further stratified by age cohorts, counties and states: (1) number of infected; (2) number of hospitalized; (3) number of ICU admissions; (4) number of deaths; and (5) hospital utilization considering available general and ICU beds in different counties.

### Effective reproduction number

Since a population will rarely be totally susceptible to an infection in the real world, the effective reproduction number, *RE*_*t*_, and not the basic reproduction number, *R*_*0*_*,* should be used as a measure of disease transmissibility at time *t*^25^. *RE*_*t*_ represents the expected number of new infections caused by an infectious individual in a population where some individuals may no longer be susceptible. Estimates of *RE*_*t*_ are typically used to assess how changes in policy, population immunity, and population behaviors, among other factors, have affected transmission at specific points in time^26–29^.

Using observed number of daily cases of COVID-19 in county *j*, we calculated timeseries of *RE*_*t,j*_ based on the methodology discussed in Cori et al. and implemented in the R-package *EpiEstim*^30^*.* This package implements a Bayesian approach for quantifying transmissibility over time during an epidemic and reports a 95% confidence interval for *RE*_*t*_. More specifically, it allows estimating the instantaneous and case reproduction numbers during an epidemic for which a timeseries of incidence is available and the distribution of the serial interval (time between symptoms onset in a primary case and symptoms onset in secondary case) is more or less precisely known. To calculate *RE*_*t,j*_, we assumed the median, mean, and standard deviation of the serial interval were 4.0, 4.7, and 2.9 days, respectively^31^. *RE*_*t,j*_ was calculated as:

10 $$RE_{t,j} = \left\{ {\begin{array}{*{20}l} {RE_{t,j}^{*} ;} \hfill & {t_{0,j} \le t \le t_{EC,j} } \hfill \\ {\max \left( {0.3,\exp \left( {\beta_{0,j} + \beta_{1,j} \times t} \right)} \right);} \hfill & {t > t_{EC,j} } \hfill \\ \end{array} } \right.$$

where *t*_*0,j*_ represents time associated with the first observed case of illness in county *j*, *t*_*EC,j*_ represents time associated with the end of model calibration period in county *j* (i.e., the last date with observed case of illness), and $RE_{t,j}^{*}$ represents output from the *EpiEstim* package. $\beta_{0,j}$ and $\beta_{1,j}$ are coefficients from fitting an exponential regression model to the estimated $RE_{t,j}^{*}$ values in the last two weeks, assuming that $RE_{t,j}^{*}$ continue the same trend observed in the past two weeks. The minimum value of 0.3 represents the estimated reproduction number in the City of Wuhan after the lockdown of the region^26^.

### Calibration of model parameters

Model calibration is the process of identifying the model parameter configurations that best explain the observed real-time values (e.g., observed cases of illness). While simple models with fewer parameters can be potentially calibrated by manually adjusting parameter values, calibration of complex models, such as M^2^-CDRM, requires extensive computational effort and resources. We used a simulation-based “optimization” method to calibrate selected model parameters by estimating their values and plausible ranges such that the model outcomes would closely match existing historic data of number of observed cases of illness.

The optimization engine in AnyLogic automatically finds the best values for different model parameters with respect to certain pre-defined constraints and requirements using the OptQuest Engine that incorporates metaheuristics to guide its search algorithm toward better solutions^32^. Inputs selected for model calibration including their ranges of plausible values are listed in Table 1. We performed the model calibration at both state and individual county levels by matching the number of reported cases of COVID-19 with model predictions, while defining constraints with respect to the expected number of deaths in the state (or individual counties). Considering October 31st, 2020 as the model training end date, we used a weighted L_1_ norm equation as:

11 $${\varvec{d}}\left( {{\varvec{X}},{\varvec{Y}}} \right) = \user2{ }\frac{{\mathop \sum \nolimits_{{{\varvec{i}} = 1}}^{{\varvec{T}}} {\varvec{\alpha}}^{{{\varvec{T}} - {\varvec{i}}}} \left| {{\varvec{X}}_{{\varvec{i}}} - {\varvec{Y}}_{{\varvec{i}}} } \right|}}{{\mathop \sum \nolimits_{{{\varvec{i}} = 1}}^{{\varvec{T}}} {\varvec{\alpha}}^{{{\varvec{T}} - {\varvec{i}}}} \times {\varvec{X}}_{{\varvec{i}}} }}$$

where **Y** = {Y_i_} (i = 1,…,T) is the target timeseries until day T (i.e., October 31st, 2020), **X** = {X_i_} (i = 1,…,T) is the model output (i.e., number of cases of illness) and a is the decay factor. We used a = 0.4 in the model optimization as reported by Venkatramanan et al.^33^.

**Table 1:** Parameters used in model calibration and their plausible range of values.

Model parameter	Description	Range of values
MIP	Mild infection period prior to recovery (days)	2–14
MIP_H_	Mild infection period prior to hospitalization (days)	2–14
IP	Incubation period (days)	1–14
CR	Critical infection rates for different age cohorts (%)^a^	1–95
URF	Under-reporting factor	1–10

^a^Constraints were defined for critical infection rates for different age cohorts as: *CR*_*0-17*_ < *CR*_*18-44*_ < *CR*_*45-64*_ < *CR*_*65-74*_ < *CR*_*75*+_.

### Multi-criteria framework for prioritizing counties based on the perceived risk of COVID-19

We used an MCDA framework to generate risk maps for individual states that highlight counties where surveillance and disease control measures could be potentially targeted based on the perceived levels of COVID-19 risks. The methodological steps required in our MCDA approach encompassed: (i) selection of decision criteria; (ii) definition of criterion measures; (iii) definition of scores assigned to each decision criterion representing low (1), medium (3), and high (9) perceived levels of risk; (iv) attribution of weights to decision criteria and (v) aggregation of risk scores across all selected decision criteria to generate the spatial maps for perceived levels of risk in each state.

Decision criteria, measures, and risk scores for ranking individual counties in each state are provided in Table 2 and briefly discussed in the following.

- *New daily cases (NDC)* this criterion, comparable to incidence in epidemiology represents the incident number of COVID-19 cases in a community. We considered a three-day average of the predicted new cases (across all age cohorts) and a cut-off value of less than *five* new cases per 100,000 residents to score this criterion. A risk score of low (1), medium (3), or high (9) was assigned to this criterion in each county if the cut-off value was met within 21 days since the training end date (October 31st, 2020), after 21 days since the training end date but before the end of the simulation period (December 31st, 2020), or was never met during the simulation period, respectively.
- *Decline in new daily deaths (NDD)* we assumed that a county must experience a sustained decline in the three-day rolling average of predicted daily hospital deaths over the course of a 21-day period to be considered low risk. Alternatively, counties that have seen few COVID cases overall would satisfy this metric if the three-day rolling average of daily new hospital deaths has never exceeded one. We used three-day average of the projected number of deaths across all age cohorts in each county and scored the county as low (1), medium (3), or high (9) if the cut-off value was met within 21 days since the training end date, after 21 days since the training end date but before the end of the simulation period, or was never met during the simulation period, respectively.
- *New hospitalizations (NH)* In addition to monitoring the decline in disease trajectory, it is important to monitor the absolute level of infection in each county. It is possible for a county that has seen a high level of infections to see a sustained decline in hospitalizations and deaths over a 21-day period while still having an underlying infection rate that is too high. Using the total number of projected new hospitalization cases across all age cohorts, each county needed to have fewer than two new hospitalizations per 100,000 residents to be considered low risk. We used three-day average of the projected number of new hospitalizations across all age cohorts in each county and scored the county as low (1), medium (3), or high (9) if the cut-off value was met within 21 days since the training end date, after 21 days since the training end date but before the end of the simulation period, or was never met during the simulation period, respectively.
- *ICU bed utilization (BU)* It is critical that regional healthcare systems have sufficient capacity for ICU beds. Taking into account the projected number of critically infected patients in each county across all ages and the ICU bed capacity in each county, we scored each county as low (1), medium (3), or high (9) if the cut-off value of 50% was met within 21 days since the training end date, after 21 days since the training end date but before the end of the simulation period, or was never met during the simulation period, respectively.

**Table 2:** Decision criteria, measures, and risk scores for ranking individual counties in each state.

Decision criterion	Criterion measure	Criterion risk scores		
		Low (1)	Medium (3)	High (9)
Three-day rolling average of new cases	< 5/100K population	Criterion met within 21 days since the training end date	Criterion met before the end of the simulation	Criterion not met before the end of the simulation
Three-day rolling average of new deaths	< 1			
Three-day rolling average of new hospitalizations	< 2/100K population			
ICU bed utilization	< 50%			

To simplify the scoring approach, we assigned equal weights to selected decision criteria and calculated aggregate risk scores across all decision criteria for different counties (RS_i_):

12 $$RS_{i} = NDC_{i} + NDD_{i} + NH_{i} + BU_{i}$$

### Summary of the model inputs

Data used in M^2^-CDRM came from a variety of sources, grouped into three categories of disease impact, demographic data, and hospital resources. Summary data used in the model, including data sources is listed in Table 3.

**Table 3:** Data used in M^2^-CDRM including their sources.

Data element	Data application	Reference
**Disease impact**		
Number of observed daily cases in different counties	Compared to predicted number of cases in different counties during the model calibration step	USA Facts: https://usafacts.org
Number of daily deaths in different counties	Used as constraints during model calibration based on the observed vases of illness in different counties	USA Facts: https://usafacts.org
**Demographic data**		
County-level population density and age distribution	Used to initialize the compartmental models for selected age cohorts	Census Bureau: https://www.census.gov/programs-surveys/decennial- census/data/datasets.2010.html
**Hospital resources**		
Age-specific hospitalization rates	Used in the disease transmission model for each age cohort	CDC: https://www.cdc.gov/coronavirus/2019-ncov/covid-data/covidview/index.html#hospitalizations
Number of general and ICU beds	Numbers of general and ICU beds adjusted by the available occupancy rates were used to calculate ICU and hospital utilization rates in different counties. Once ICU capacity is reached in a county, new patients in need of ICU admission would be transferred to the deceased population compartment (D_i,j_)	Centers for Medicare & Medicaid Services’ Healthcare Cost Report Information System (HCRIS): https://www.cms.gov/Research-Statistics-Data-and-Systems/Statistics-Trends-and-Reports/Medicare-Provider-Cost-Report
Hospital occupancy rates	State-level acute care and critical access hospital occupancy rates in urban vs rural areas were used to adjust number of available general and ICU beds available in each county	American hospitals directory: https://www.ahd.com/news/HFM_DataTrends_2018_July.pdf

## Results

### State-level predictions

Tables 4 and 5 summarize the model predictions for number of COVID-19 cases aggregated across all age cohorts in the 20 most populous states in the United States. We reported a range of values for two-week (November 14, 2020), three-week (November 21, 2020), and four-week (November 28, 2020) out-of-sample model predictions based on the 95% confidence intervals reported for *RE*_*t*_. We also reported the cumulative observed values for COVID-19 cases by selected dates and % error calculated by comparing the observed values with mean predictions. For each of these states, selected model parameters (listed in Table 1) were calibrated to replicate the observed cumulative number of cases between March 15 and October 31, 2020 across the whole state. We further used the state-wide calibrated model parameters for all individual counties in the selected state assuming no change in disease epidemiology in different localities (e.g., no change in critical infection rate for a particular age cohort across different counties in California). Summary results typically showed underestimated number of COVID-19 cases with variability in % error across different states. Furthermore, we observed relative decrease in model accuracy when period of out-of-sample predictions was increased from two to four weeks. For example, average % error for two-week out-of-sample prediction of cases was − 6.7% across all 20 states with a range of values between − 1.1% (California) and -16.9% (Michigan). We observed lower accuracy for the four-week out-of-sample case predictions with an average % error value of − 16.2% across all 20 states and a range of values between − 7.1 and − 32.4% for California and Michigan, respectively. Model results showed similar patterns for predicted number of COVID-19 deaths across these selected states (Table 5); however, the prediction accuracies were typically higher for cumulative number of deaths by selected dates. For example, average % error for two-week out-of-sample prediction of deaths across selected states was 3.2% (compared to − 6.7% error for prediction of cases) with a range of values between 0.1 and 15.2% for Missouri and Washington, respectively.

**Table 4:** Model performance for two-, three-, and four-week out-of-sample predictions of the cumulative COVID-19 cases in the top 20 populous states.

State	Two-week out-of-sample predictions (November 14, 2020)			Three-week out-of-sample predictions (November 21, 2020)			Four-week out-of-sample predictions (November 28, 2020)		
	Range of predictions	Observed	% Error	Range of predictions	Observed	% Error	Range of predictions	Observed	% Error
California	964,486–1,017,792	990,096	− 1.1	991,330–1,101,984	1,053,945	− 3.3	1,021,323–1,211,919	1,147,417	− 7.1
Texas	927,085–1,044,511	984,377	− 3.0	944,996–1,152,937	1,050,255	− 5.0	963,036–1,275,089	1,128,131	− 7.6
Florida	819,518–844,319	852,174	− 2.7	836,321–882,500	897,322	− 4.9	853,484–927,956	953,300	− 7.7
New York	515,129–543,158	536,214	− 2.3	521,856–573,152	568,847	− 5.6	528,035–608,774	607,070	− 9.4
Pennsylvania	215,722–232,495	238,657	− 7.0	223,702–254,648	275,513	− 14.9	231,659–281,377	321,070	− 22.7
Illinois	419,938–459,360	511,169	− 15.2	436,352–508,202	597,818	− 23.0	452,833–565,200	674,072	− 27.5
Ohio	221,957–242,352	261,483	− 12.1	230,590–267,693	305,365	− 20.1	239,068–297,812	371,908	− 30.3
Georgia	347,637–370,294	376,032	− 5.8	351,721–391,865	391,429	− 7.4	355,332–416,164	408,643	− 9.3
North Carolina	278,608–295,263	297,973	− 4.4	284,890–314,174	316,955	− 6.9	290,521–334,812	343,408	− 11.0
Michigan	195,442–218,456	245,252	− 16.9	204,300–246,177	296,840	− 26.3	213,149–279,695	347,746	− 32.4
New Jersey	245,806–255,510	260,430	− 4.1	253,457–271,881	285,519	− 8.7	261,572–292,107	313,863	− 13.1
Virginia	184,386–197,766	194,906	− 3.0	187,695–210,732	206,751	− 5.5	190,612–224,668	223,568	− 9.9
Washington	108,774–115,661	120,011	− 7.5	110,811–123,182	134,118	− 14.6	112,670–131,872	151,018	− 21.9
Arizona	249,274–255,512	263,133	− 4.3	253,739–265,544	279,896	− 7.8	258,348–277,724	306,868	− 13.6
Massachusetts	168,537–179,938	180,753	− 4.9	173,406–198,166	197,561	− 9.0	178,620–224,517	214,874	− 11.9
Tennessee	268,495–293,014	289,749	− 4.2	279,257–322,714	320,729	− 7.9	289,913–356,839	345,853	− 9.0
Indiana	186,156–203,048	222,186	− 13.3	193,852–224,421	265,099	− 22.6	201,574–250,066	309,503	− 29.3
Missouri	190,799–207,726	220,768	− 10.6	198,389–228,183	253,473	− 17.3	205,381–250,666	282,792	− 21.6
Maryland	148,742–159,294	156,709	− 2.9	151,885–170,824	169,804	− 7.1	154,884–184,520	185,464	− 11.8
Wisconsin	256,093–279,759	293,812	− 9.5	275,475–320,478	342,155	− 14.2	295,568–369,722	386,441	− 16.1

**Table 5:** Model performance for two-, three-, and four-week out-of-sample predictions of the cumulative COVID-19 deaths in the top 20 populous states.

State	Two-week out-of-sample predictions (November 14, 2020)			Three-week out-of-sample predictions (November 21, 2020)			Four-week out-of-sample predictions (November 28, 2020)		
	Range of predictions	Observed	% Error	Range of predictions	Observed	% Error	Range of predictions	Observed	% Error
California	18,684–19,020	18,069	4.0	19,211–20,055	18,356	6.0	19,737–21,493	18,876	6.9
Texas	18,973–19,734	18,850	1.9	19,516–21,245	19,680	1.5	19,959–23,236	20,736	0.3
Florida	16,925–17,049	17,248	− 1.5	17,295–17,586	17,643	− 1.3	17,659–18,202	18,157	− 1.6
New York	32,652–33,326	33,486	− 1.7	33,296–34,747	33,690	0.4	33,867–36,471	33,961	2.2
Pennsylvania	10,176–10,387	9,086	13.0	10,630–11,088	9,355	15.6	11,070–11,919	9,951	14.5
Illinois	10,301–10,613	10,289	1.3	10,824–11,511	10,874	1.9	11,317–12,585	11,677	0.9
Ohio	5,575–5,696	5,547	1.4	5,858–6,117	5,742	3.8	6,142–6,618	6,118	3.3
Georgia	8,276–8,403	8,259	0.8	8,444–8,740	8,481	0.5	8,577–9,129	8,641	1.3
North Carolina	4,202–4,279	4,638	− 8.9	4,368–4,517	4,719	− 6.4	4,516–4,780	5,039	− 8.4
Michigan	8,457–8,765	8,093	6.0	8,950–9,635	8,510	8.1	9,415–10,677	9,094	8.6
New Jersey	17,227–17,434	16,461	5.2	17,713–18,155	16,618	7.7	18,204–19,027	16,819	10.2
Virginia	3,991–4,057	3,717	7.9	4,138–4,305	3,827	9.9	4,276–4,558	3,973	10.1
Washington	2,836–2,888	2,479	15.2	2,925–3,043	2,566	15.8	2,999–3,222	2,680	14.7
Arizona	6,021–6,069	6,192	− 2.4	6,130–6,235	6,312	− 2.2	6,238–6,435	6,513	− 3.1
Massachusetts	11,279–11,526	10,184	11.6	11,588–12,189	10,360	13.7	11,907–13,181	10,551	16.3
Tennessee	3,884–3,975	3,670	6.9	4,080–4,275	3,994	3.8	4,272–4,629	4,372	1.0
Indiana	4,856–4,959	4,731	3.7	5,112–5,329	5,024	3.5	5,356–5,756	5,435	1.5
Missouri	3,287–3,371	3,321	0.1	3,507–3,692	3,474	3.2	3,703–4,059	3,774	1.9
Maryland	4,463–4,551	4,279	5.0	4,593–4,791	4,379	6.5	4,719–5,064	4,519	7.1
Wisconsin	2,223–2,288	2,395	− 6.1	2,442–2,569	2,739	− 8.9	2,646–2,926	3,114	− 11.1

### County-level predictions

For each of the state-level predictions listed in Tables 4 and 5, our model generated results for each individual county within a state, allowing for analysis of the heterogenous disease growth patterns across localities. Although each county used an independent predicted timeseries for *RE*_*t*_ based on the county-specific observed cases of illness, a simplifying assumption was made that calibrated disease parameters (listed in Table 1) were homogenous across all counties in a particular state when model was trained to replicate the state-level observed cumulative number of cases and deaths between March 15, 2020–October 31, 2020. We further investigated the impact of this assumption on the model prediction accuracy by conducting a county-level calibration experiment across three localities in Virginia, including Richmond City, Montgomery County, and Norfolk City. The experiment included two scenarios to evaluate the out-of-sample model prediction accuracy between November 1 and 28 based on: (1) calibrated model parameters using state-level observed data (223,568 and 3973 for observed cumulative cases of illness and deaths in Virginia, respectively); and (2) county-level calibrated model parameters based on the county-specific observed data (6606, 3884, and 6423 for observed cases and 82, 15, and 89 for observed deaths in Richmond City, Montgomery County, and Norfolk City, respectively).

**Figure 3:**
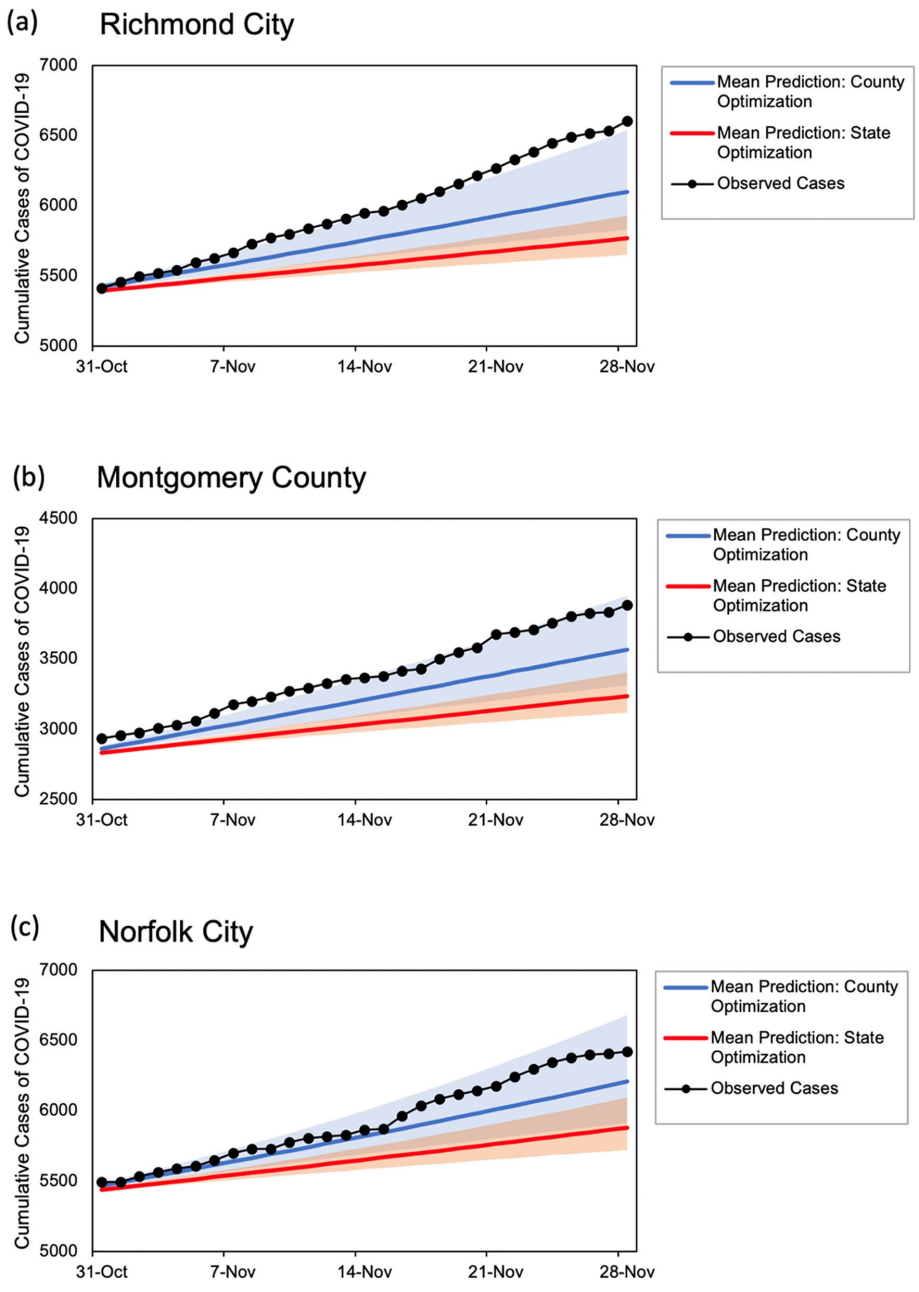
COVID-19 case projection comparison between state and county optimization for three localities in Virginia: (**a**) Richmond City; (**b**) Montgomery County; and (**c**) Norfolk City.

Figure 3 shows the resulting timeseries for the out-of-sample model predictions between November 1 and 28, 2020 for selected localities in Virginia including cumulative number of observed COVID-19 cases during the same time period. Each predicted timeseries represents model results for the cumulative COVID-19 cases based on the mean *RE*_*t*_ value as well as range of cases based on the 95% confidence interval associated with *RE*_*t*_ (shaded areas). Results indicated that conducting county-level model calibration led to increase in model accuracy. For example, % errors for four-week out-of-sample predictions were − 11.3%, − 15.4%, and − 8.4% for Richmond City, Montgomery County, and Norfolk City, respectively, when model parameters were calibrated using state-level cumulative number of observed cases. When model parameters were calibrated for each individual county, % errors reduced to − 6.9%, − 7.8%, and − 4.0% for the selected counties.

### State-level risk maps using MCDA

**Figure 4:**
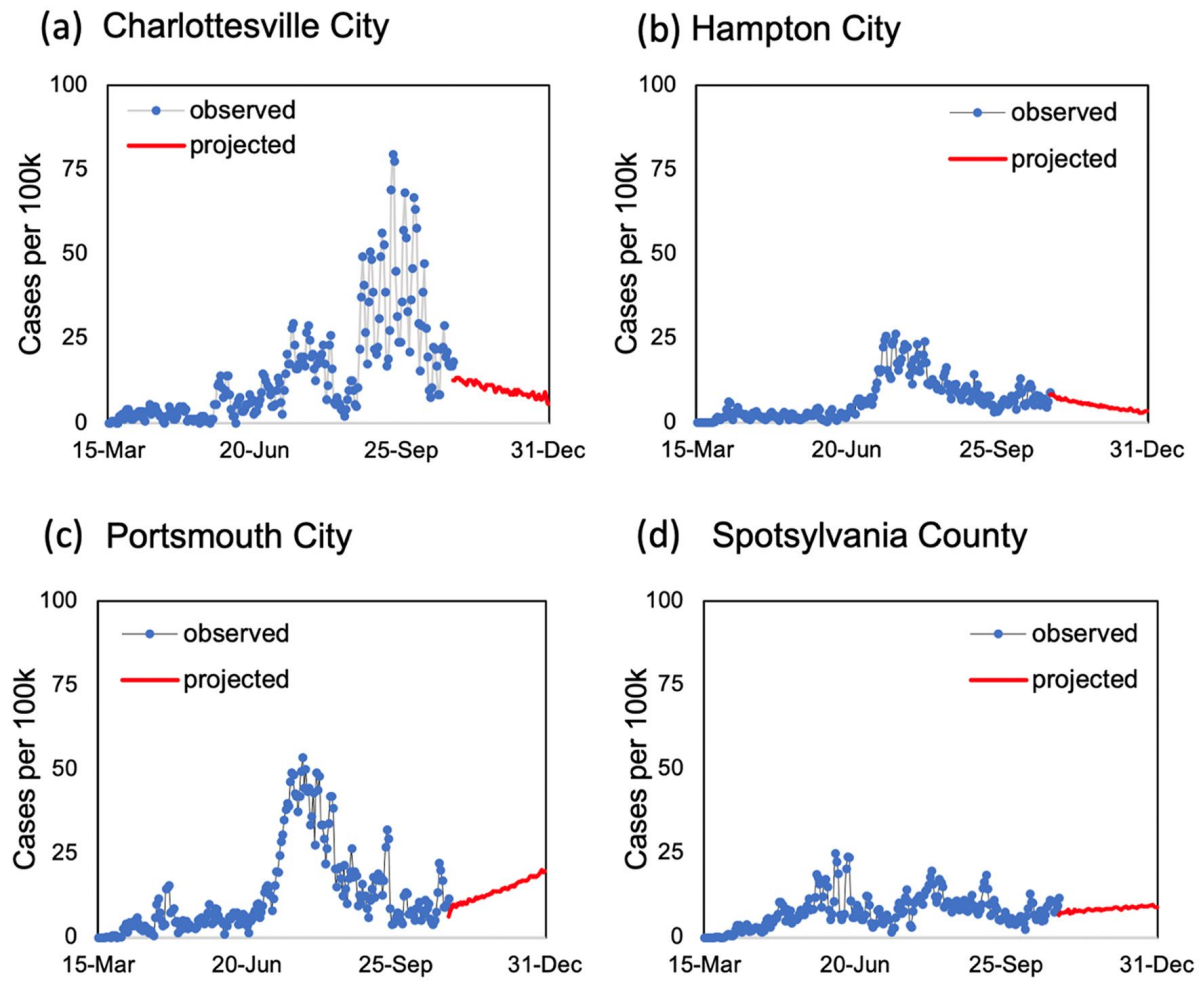
Three-day rolling average of new COVID-19 cases per 100,000 residents estimated based on the mean estimated *RE*_*t*_ values for four localities in Virginia: (**a**) Charlottesville City, (**b**) Hampton City, (**c**) Portsmouth City, and (**d**) Spotsylvania County.

**Figure 5:**
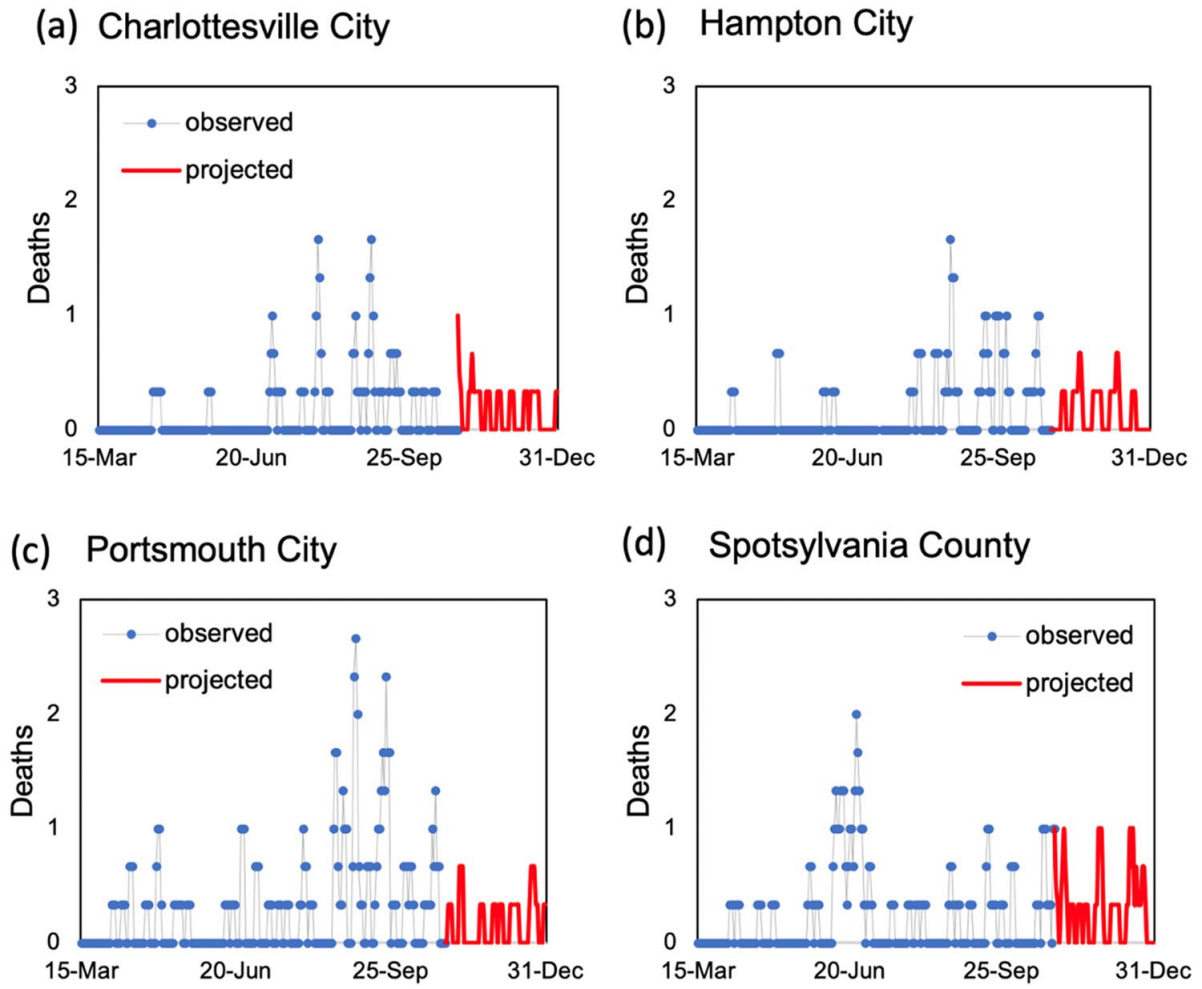
Three-day rolling average of new COVID-19 deaths based on the mean estimated *RE*_*t*_ values for four localities in Virginia: (**a**) Charlottesville City, (**b**) Hampton City, (**c**) Portsmouth City, and (**d**) Spotsylvania County.

**Figure 6:**
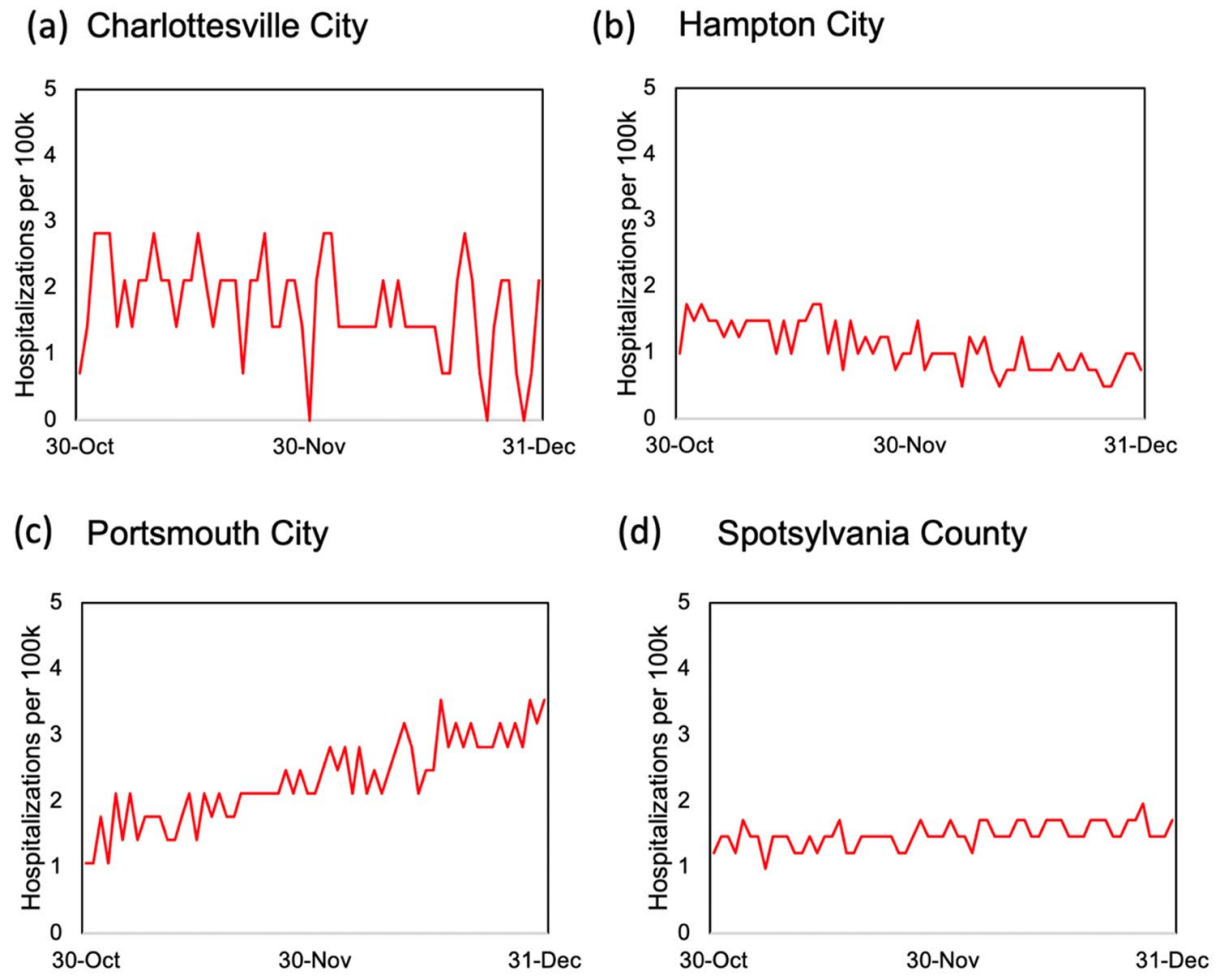
Three-day average of new COVID-19 hospitalizations per 100,000 persons projections based on the mean estimated *RE*_*t*_ values for four localities in Virginia: (**a**) Charlottesville City, (**b**) Hampton City, (**c**) Portsmouth City, and (**d**) Spotsylvania County.

**Figure 7:**
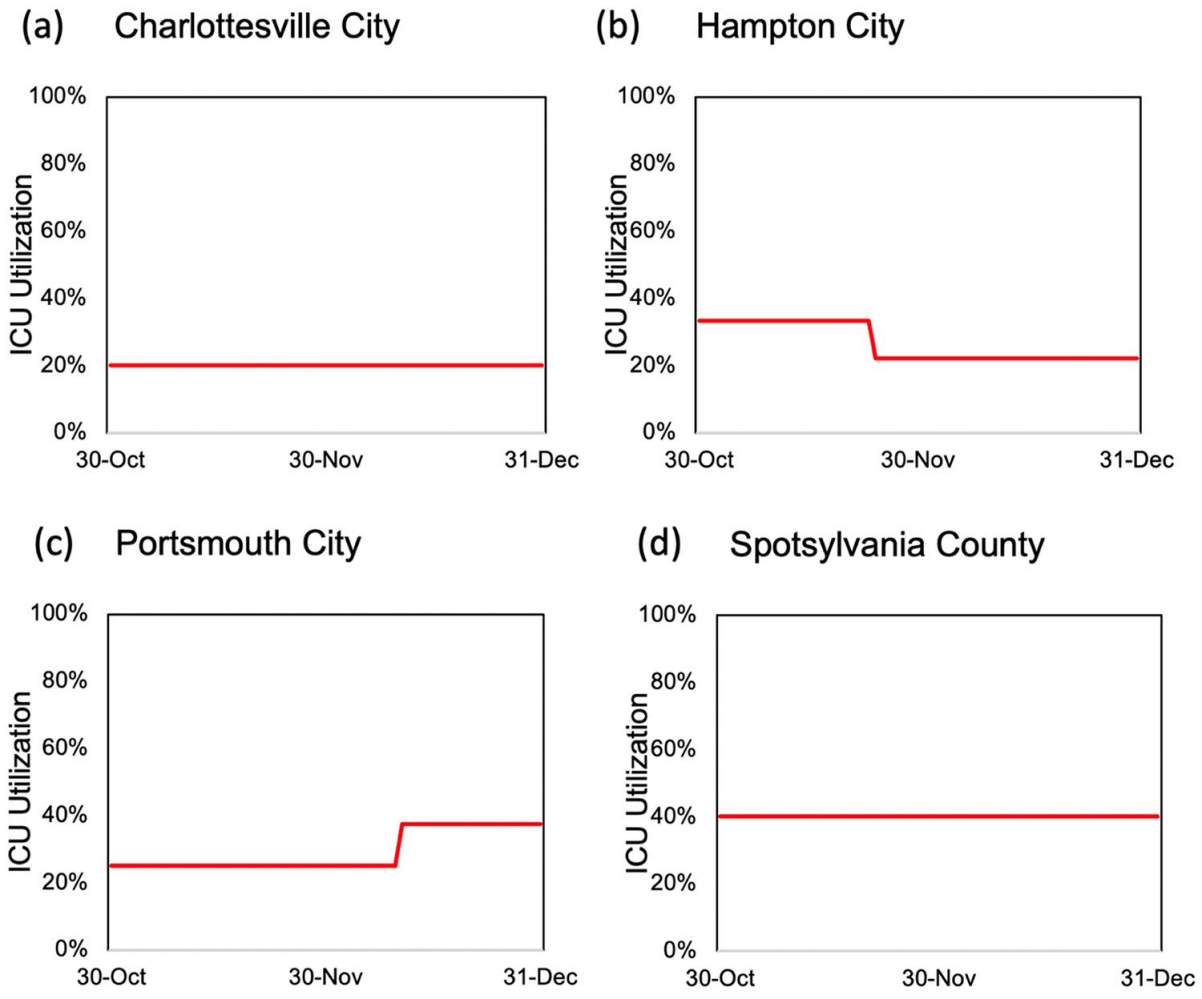
COVID-19 ICU bed utilization projections based on the mean estimated *RE*_*t*_ values for four localities in Virginia: (**a**) Charlottesville City, (**b**) Hampton City, (**c**) Portsmouth City, and (**d**) Spotsylvania County.

In addition to out-of-sample case and death predictions across different localities in individual states, we utilized various county-level model outputs, including three-day rolling average of new daily cases per 100,000 residents, three-day rolling average of daily new hospital deaths, three-day rolling average of new hospitalizations per 100,000 residents, and ICU bed utilization percentages, and time to meet their cut-off values (listed in Table 2) to score individual counties with respect to their perceived levels of COVID-19 risks. Examples of model outputs for selected decision criteria are shown in Figs. 4,5,6 and 7 for four localities in Virginia, including Charlottesville City, Hampton City, Portsmouth City, and Spotsylvania County. Model results typically showed substantial variability in number of days required to achieve the scoring requirements for selected decision criteria since the training end date (October 31, 2020). For example, for the counties that have not met the criterion requirement before October 31, number of days to achieve a three-day rolling average of new cases per 100,000 residents of five or less was 59.7 days on average with a minimum value of only two days for Norton City while 85 out of 133 counties (64%) did not satisfy this requirement by the end of the model simulation time of December 31, 2020 (data not shown here).

**Figure 8:**
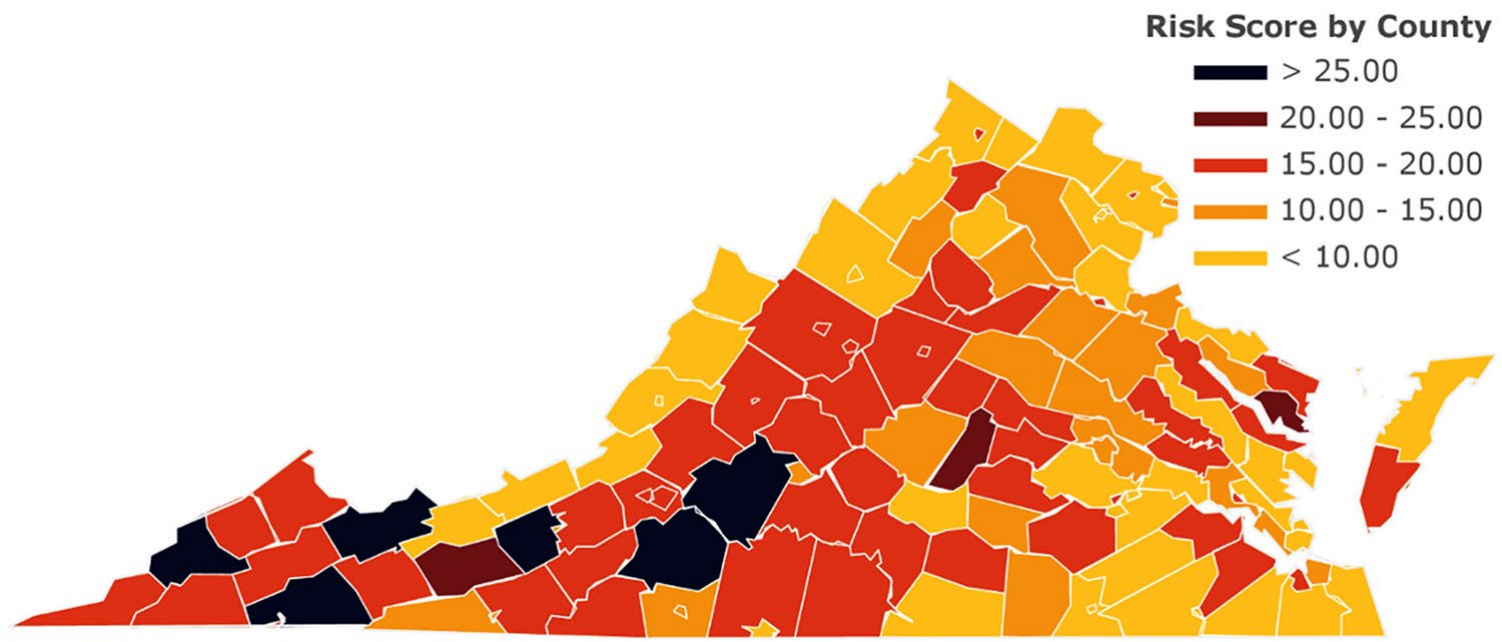
Aggregated risk scores for individual counties in Virginia.

We also calculated the aggregated risk scores across selected decision criteria for all counties in Virginia. The risk map based on the aggregated scores is shown in Fig. 8. Aggregated risk scores showed spatial variability with an average value of 14.3 across all counties and minimum and maximum values of 4 and 30, respectively. The model typically predicted higher aggregated risk scores (15 or higher) in the southwestern localities while lower scores (15 or lower) in the northern and eastern localities of the state, primarily due to additional hospital resources (e.g., number of general and ICU beds) in those counties.

## Discussion

The COVID-19 pandemic has resulted in a global health crisis with unprecedented growing economic, social, and health impacts not seen since the 1918 Spanish flu pandemic. Computational models have played an important role in the ongoing crisis by providing insights regarding the disease spread dynamics as well as the potential impacts of public policies at the local, national, and global levels. Different models with a wide range of underlying methodologies have been used by policy makers and public health officials to assess the evolution of the COVID-19 pandemic, design and analyze control measures, and study various what-if scenarios. For example, the Centers for Disease Control and Prevention (CDC) has been working with different partners to bring together weekly COVID-19 forecasts based on statistical and mathematical models aiming to predict national and state numbers of new and total COVID-19 deaths as well as cases of infection and hospitalization^34^. Table 6 provides a summary of selected COVID-19 computational models available from the CDC website including their key features, geographic scope, methodology, frequency of updates, and ability to conduct what-if scenario analysis. The majority of these models have adapted different forms of the SD-based models (e.g., SEIR) with geographical scopes typically limited to the national or state level predictions. All models faced challenges due to availability of data, rapidly evolving pandemic and unprecedented control measures put in place. Despite these challenges, we believe that mathematical models can provide useful and timely information to the policy makers.

**Table 6:** Summary of selected COVID-19 models including underlying methodologies, predicted features, spatial resolution, scenario analysis features, and frequency of data updates.

Model name	Institution	URL	Methodology	Predicted features^a^	Spatial resolution^b^	Scenario analysis	Frequency of data updates
COVID Forecast Hub	University of Massachusetts-Amherst Reich Lab	https://covid19forecasthub.org/	Ensemble method combining results from multiple models	C, D, H,	N, S, C	Selected individual models in the ensemble method include scenario analysis	Weekly
Auquan	CDC, Auquan Data Science	https://covid19-infection-model.auquan.com/	Fitted SD model (SEIR)	C, D	G, N, S	Limited to selected model parameters (e.g., infection spread, social distancing)	Daily
Columbia	Columbia Mailman School of Public Health	https://cuepi.shinyapps.io/COVID-19/	SD model (SEIR)	C, H	S, C	Limited to adjustments to the R_0_ values	Daily
Columbia-UNC	Columbia University and UNC Chapel Hill	https://github.com/COVID19BIOSTAT/covid19_prediction	Survival-convolution model	C, D	N	NA	NA
IHME	University of Washington—Institute for Health Metrics and Evaluation	https://covid19.healthdata.org/united-states-of-america?view=total-deaths&tab=trend	SD model (SEIR) calibrated using real-world data	C, D, H	G, N, S	Scenario analysis based on vaccination, mask use, and government-imposed mandates	Frequently
DDS	University of Texas at Austin UT	https://dds-covid19.github.io/index.html	Negative binomial linear dynamic system	C, D	N, S	NA	NA
Google-HSPH	Google Cloud AI	https://datastudio.google.com/c/reporting/52f6e744-66c6-47aa-83db-f74201a7c4df/page/EfwUB	Combination of SD model (SEIR) and covariates encoding within a computational graph framework	C, D, H	S, C	NA	Bi-weekly
ISU	Iowa State University	https://covid19.stat.iastate.edu/	Discrete-time spatial epidemic model	C, D	S, C	NA	Daily
JHU-APL	John Hopkins University Applied Physics Laboratory LLC	https://buckymodel.com/	Spatially distributed SD models (SEIR) stratified based on age	C, D, H	S, C	NA	NA
MIT-ORC	Massachusetts Institute of Technology Operations Research Center	https://www.covidanalytics.io/projections	Adjusted SD model (SEIR)	C, D, H	G, N, S	NA	NA
Northeastern—MOBS	Northeastern University	https://covid19.gleamproject.org/	Adjusted SD model (SEIR) using a metapopulation approach and age-specific contact matrix	C, D, H	N, S	Scenario analysis based on different levels of social distancing	Weekly
Oliver Wyman	Oliver Wyman	https://pandemicnavigator.oliverwyman.com/	Extended SD model (SIR) including detected and undetected infected populations	C, D	G, N, S, C	Scenario analysis based on mobility and testing	Daily
UCLA	University of California LA	https://covid19.uclaml.org/	Adjusted SD model (SEIR) accounting for unreported recovery	C, D	G, N, S	NA	Weekly
UCSB	University of California Santa Barbara	https://github.com/Gandor26/covid-open/	Attention crossing time series	C	S	NA	Weekly
UGA—CEID	University of Georgia Center for the Ecology of Infectious Disease	https://github.com/cdcepi/COVID-19-Forecasts/blob/master/COVID-19_Forecast_Model_Descriptions.md#Auquan	Statistical Random Walk Model	C, D	N, S, C	NA	Weekly
UT	University of Texas	https://covid-19.tacc.utexas.edu/projections/	Ensemble of curve fitting and SD model (SEIR)	D	S	NA	Daily

^a^*C* Case prediction, *D* death prediction, *H* hospitalization prediction.

^b^*G* Global-level predictions (i.e., different countries), *N* national-level predictions, *S* state-level predictions, *C* county-level predictions.

Like other computational modeling methods, commonly used SD-based models can be especially useful when invoked for the right task, however they are not appropriate for all forecasting, prediction, and scenario simulations. These models operate at an elevated level of abstraction, assume population homogeneity, and typically lack the ability to update underlying model parameters once new, real-time data become available. In this study, we developed a multi-method modeling approach by using an ABM framework to combine thousands of age-stratified and location-specific SEIR models that could potentially capture essential virus transmission dynamics for the purpose of modeling COVID-19 spread over time and in different localities with increased model fidelity. The proposed simulation model showed potential for use by decision makers as an effective virtual laboratory in performing what-if analysis and quantifying perceived levels of health risks by combining forecasted outcomes with user-defined health metrics in a multi-criteria decision framework. While the current case study is focused on COVID-19, the modular framework of our solution easily allows future adaptation to any high-consequence public health threats.

We have also addressed some of the key limitations of SD-based epidemiological models. First, current SD-based epidemiological models typically approximate the spread of COVID-19 at the state and national level. These models do not account for the effect of mitigation policies, population demographics, or cohort behaviors on disease spread dynamics at local levels. Our multi-method approach provided enhanced precision and fidelity at the local level. Second, existing SD-based models typically focus on the constant value of the basic reproduction number (R_0_) as a measure of disease transmissibility. We used potential changes in R_0_ over time, represented by R_E_, which reflected how the disease transmission within the population changed over time. We used this dynamic adjustment to assess how changes in mitigation policies, population immunity, and population behaviors, among other factors, could potentially affect COVID-19 transmission at specific time and location points. Lastly, most SD-based models fail to account for the effect of population demographics (e.g., age), particularly at the county and local levels. We believe that characterizing model parameters such as disease transmission, hospitalization, critical infection, and fatality rates based on the population demographics potentially mitigates the bias for under-represented segments of the population.

We are also aware that computational models are approximations of the real-life scenarios. There are currently no predictive models that generate a highly accurate picture of the COVID-19 disease spread or its clinical impacts, including ours, as too many factors can potentially affect the spread of the disease. For example, our model showed to underestimate cases and overestimate deaths. Modelling exercises tend to carry forward certain distortions that are inherent to the complex and dynamic characteristics of real-world reporting systems when considering rapidly evolving epidemiological scenarios. In the case of COVID-19, factors such as a sub-optimal standardization in the coding and reporting of potential, suspected, and confirmed cases may have introduced information biases in reality that generate mismatches with the model outcomes. A similar phenomenon could have taken place in terms of inaccuracies regarding causes of deaths and the role of COVID-19 in death certificates.

We also acknowledge that there were multiple sources of uncertainty in our model resulting in prediction inaccuracies and errors as reported in Tables 4 and 5. Key sources of uncertainty in our model potentially included model structure (e.g., set of differential equations identified for disease dynamics), model detail (e.g., simplifying assumptions related to reinfection as well as between-county population movements), model calibrations (e.g., state versus county-level parameter calibration), and scenario reasonableness (e.g., assumption of homogenous age-stratified reproduction numbers.

There are areas for improvement in our modeling approach that can potentially reduce the above uncertainties and enhance the prediction accuracies. For example, alternative sets of scientific or technical assumptions might be available for developing the complex dynamics of COVID-19 disease spread. The implications of these alternative foundations may be evaluated by constructing alternative models and comparing results across different solutions. It may be possible to potentially parameterize alternative model structures into a higher order model, and to evaluate the impact of modeling assumptions using sensitivity analysis. Also, while we used the observed daily cases of COVID-19 to characterize location-specific timeseries for R_E_, future values were approximated using exponential regression models fitted to the latest two weeks of data. This approximation may potentially pose bias and limitations in forecasting the disease dynamics in populous areas where changes in behaviors (e.g., lack of social distancing, limited stay-at-home restrictions) can significantly impact the disease spread trajectory. We understand that recent studies have demonstrated promising use of novel forecasting methodologies to characterize relationships between human micro-level activities and movements based on telemetry data and micro-level R_E_ values^35–37^. Such methodologies can be potentially coupled with our modeling approach. Furthermore, our model relies on the current body of evidence with regards to the chances of reinfection. In this sense, recovered patients are considered to be immune to future COVID-19 infections. These assumptions are being revised as new viral variants are identified, which might imply the need to redefine the basic assumptions of the model. Also, the current approach for calibrating the model parameters is largely an ad-hoc simulation-based procedure based on the state-level observed cases of infection as well as death. Although computationally intensive, we demonstrated that the model accuracy could be substantially improved when calibrations were conducted at the local levels (e.g., individual counties). Finally, we did not estimate age-stratified timeseries for R_E_ because reported daily cases of COVID-19 currently do not contain demographic data including age. Accounting for heterogeneity in transmission due to demographic factors and also estimating age-stratified reproduction numbers could provide insight into differences in transmission potential by age and other factors. In addition, although the use of age serves as proxy of several risk factors and health conditions, subsequent improvements of this modeling approach could account for other epidemiological and demographic population characteristics that are highly correlated with COVID-19 transmission and outcomes. This is the case for co-morbidities, mobility patterns, population density, and climate, among others.

## References

[1] Lang T (2020). Plug COVID-19 research gaps in detection, prevention and care. Nature.

[2] Bhatia, R., Sledge, I. & Baral, S. The missing science: Epidemiological data gaps for COVID-19 policy in the United States. *medRxiv.*10.1101/2021.02.11.21251602 (2021).

[3] Jenner AL, Aogo RA, Davis CL, Smith AM, Craig M (2020). Leveraging computational modeling to understand infectious diseases. Curr. Pathobiol. Rep..

[4] Kok S (2015). Optimizing an HIV testing program using a system dynamics model of the continuum of care. Health Care Manag. Sci..

[5] Thompson KM, Duintjer Tebbens RJ, Pallansch MA, Wassilak SG, Cochi SL (2015). Polio eradicators use integrated analytical models to make better decisions. Interfaces.

[6] Sharareh, N., Sabounchi, N.S., Sayama, H. & MacDonald, R. The Ebola crisis and the corresponding public behavior: a system dynamics approach. *PLoS Curr.* **8** (2016). 10.1371/currents.outbreaks.23badd9821870a002fa86bef6893c01d PMC5118047 27974995

[7] van Ackere A, Schulz PJ (2020). Explaining vaccination decisions: a system dynamics model of the interaction between epidemiological and behavioral factors. Socio-Econ. Plan. Sci..

[8] Anderson RM, May RM (1991). Infectious Diseases of Humans: Dynamics and Control.

[9] Keeling MJ, Rohani P (2011). Modeling Infectious Diseases in Humans and Animals.

[10] Roberts M, Andreasen V, Lloyd A, Pellis L (2015). Nine challenges for deterministic epidemic models. Epidemics.

[11] Merler S (2015). Spatiotemporal spread of the 2014 outbreak of Ebola virus disease in Liberia and the effectiveness of non-pharmaceutical interventions: a computational modelling analysis. Lancet Infect. Dis..

[12] Crooks AT, Hailegiorgis AB (2014). An agent-based modeling approach applied to the spread of cholera. Environ. Modell. Softw..

[13] Macal, C. M. & North, M. J. (2005). Tutorial on agent-based modeling and simulation. *Proceedings of the Winter Simulation Conference, IEEE*. 10.1109/WSC.2005.1574234 (2005).

[14] Crooks, A. T. & Heppenstall, A. J. Introduction to Agent-Based Modeling. *in Agent-based models of geographical systems* 85–105 (Springer, Netherlands, 2012).

[15] Midgley D, Marks R, Kunchamwar D (2007). Building and assurance of agent-based models: an example and challenge to the field. J. Bus. Res..

[16] Bonabeau E (2002). Agent-based modeling: methods and techniques for simulating human systems. PNAS.

[17] Frias-Martinez, E., Williamson, G. & Frias-Martinez, V. An agent-based model of epidemic spread using human mobility and social network information. In *2011 IEEE 3*^*rd*^* international conference on privacy, security, risk and trust and 2011 IEEE third international conference on social computing*, IEEE. 10.1109/PASSAT/SocialCom.2011.142 (2011).

[18] Li, Y., Zhang, Y. & Cao, L. Evaluation and selection of hospital layout based on an integrated simulation method. *WSC,* 2560–2568 (2020).

[19] Brailsford, S.C. Hybrid simulation in healthcare: new concepts and new tools. *WSC*, 1645–1653 (2015).

[20] Viana, J. Reflections on two approaches to hybrid simulation in healthcare. *WSC,* 1585–1596 (2014).

[21] Vaughan L (2021). Relationship of socio-demographics, comorbidities, symptoms and healthcare access with early COVID-19 presentation and disease severity. BMC Infect. Dis..

[22] Davies NG (2020). Age-dependent effects in the transmission and control of COVID-19 epidemics. Nat. Med..

[23] Lau MS (2020). Characterizing superspreading events and age-specific infectiousness of SARS-CoV-2 transmission in Georgia, USA. PNAS.

[24] Wu SL (2020). Substantial underestimation of SARS-CoV-2 infection in the United States. Nat. Commun.

[25] Gostic KM (2020). Practical considerations for measuring the effective reproductive number, R_t_. medRxiv.

[26] Pan A (2020). Association of public health interventions with the epidemiology of the COVID-19 outbreak in Wuhan, China. JAMA.

[27] Kucharski AJ (2020). Early dynamics of transmission and control of COVID-19: a mathematical modeling study. Infect. Dis..

[28] Cauchemez S, Kiem CT, Paireau J, Rolland P, Fontanet A (2020). Lockdown impact on COVID-19 epidemics in regions across metropolitan France. Lancet.

[29] Flaxman S (2020). Estimating the effects of non-pharmaceutical interventions on COVID-19 in Europe. Nature.

[30] Cori A, Ferguson NM, Fraser C, Cauchemez S (2013). A new framework and software to estimate time- varying reproduction numbers during epidemics. Am. J. Epidemiol..

[31] Nishiura H (2020). Correcting the actual reproduction number: a simple method of estimating R0 from early epidemic growth data. Int. J. Environ. Res. Public Health.

[32] Laguna M (1997). Optimization of Complex Systems with OptQuest.

[33] Venkatramanan S (2018). Using data-driven agent-based models for forecasting emerging infectious diseases. Epidemics.

[34] Center for Disease Control and Prevention. Interpretation of forecasts of new and total deaths. *CDC* https://www.cdc.gov/coronavirus/2019-ncov/covid-data/forecasting-us.html (2021).

[35] Rudiger, S. *et al.* Forecasting the SARS-CoV-2 effective reproduction number using bulk contact data from mobile phones. *medRxiv*. 10.1101/2020.10.02.20188136 (2020). 10.1073/pnas.2026731118 PMC8346907 34261775

[36] Linka, K., Goriely, A. & Kuhl, E. Global and local mobility as a barometer for COVID-19 dynamics. *medRxiv*. 10.1101/2020.06.13.20130658 (2020). 10.1007/s10237-020-01408-2 PMC7809648 33449276

[37] Leung, K., Wu, J.T. & Leung G.M. Real-time tracking and prediction of COVID-19 infection using digital proxies of population mobility and mixing. *medRxiv*. 10.1101/2020.10.17.20214155 (2020). 10.1038/s41467-021-21776-2 PMC7940469 33686075

